# The association of depression and anxiety with cardiac autonomic activity: The role of confounding effects of antidepressants

**DOI:** 10.1002/da.22966

**Published:** 2019-10-17

**Authors:** Mandy X. Hu, Yuri Milaneschi, Femke Lamers, Ilja M. Nolte, Harold Snieder, Conor V. Dolan, Brenda W. J. H. Penninx, Eco J. C. de Geus

**Affiliations:** ^1^ Department of Psychiatry, Amsterdam Public Health Research Institute, Amsterdam UMC Vrije Universiteit Amsterdam Amsterdam The Netherlands; ^2^ Department of Epidemiology, Medical Center Groningen University of Groningen Groningen The Netherlands; ^3^ Department of Biological Psychology VU University Amsterdam The Netherlands

**Keywords:** antidepressants, anxiety/anxiety disorders, depression, electrophysiology, gene‐environment, genetics, stress

## Abstract

**Background:**

Depression and anxiety may unfavorably impact on cardiac autonomic dysregulation. However, it is unclear whether this relationship results from a causal effect or may be attributable to confounding factors. We tested the relationship between depression and anxiety with heart rate (HR) and heart rate variability (HRV) across a 9‐year follow‐up (FU) period and investigated possible confounding by antidepressant use and genetic pleiotropy.

**Methods:**

Data (no. of observations = 6,994, 65% female) were obtained from the longitudinal Netherlands Study of Depression and Anxiety, with repeated waves of data collection of HR, HRV, depression, anxiety, and antidepressant use. Summary statistics from meta‐analyses of genome‐wide association studies were used to derive polygenic risk scores of depression, HR, and HRV.

**Results:**

Across the 9‐year FU, generalized estimating equations analyses showed that the relationship between cardiac autonomic dysregulation and depression/anxiety rendered nonsignificant after adjusting for antidepressant use. A robust association was found between antidepressant use (especially tricyclic antidepressants, selective serotonin, and noradrenalin reuptake inhibitors) and unfavorable cardiac autonomic activity across all waves. However, no evidence was found for a genetic correlation of depression with HR and HRV, indicating that confounding by genetic pleiotropy is minimal.

**Conclusions:**

Our results indicate that the association between depression/anxiety and cardiac autonomic dysregulation does not result from a causal pathway or genetic pleiotropy, and these traits might therefore not be inevitably linked. Previously reported associations were likely confounded by the use of certain classes of antidepressants.

## 1 INTRODUCTION

Depressive and anxiety disorders have been found to be associated with cardiac autonomic dysregulation, reflected by a significantly higher heart rate (HR) and lower heart rate variability (HRV) than healthy controls (Alvares, Quintana, & Hickie, 2016; Bleil, Gianaros, Jennings, Flory, & Manuck, 2008; Carney, Freedland, & Veith, 2005; Chalmers, Quintana, Abbott, & Kemp, 2014; Chang et al., 2012; Chang et al., 2013; Hu et al., 2018; Kemp, Quintana, Felmingham, Matthews, & Jelinek, 2012; Watkins, Grossman, Krishnan, & Sherwood, 1998; Yeh et al., 2016). This correlational finding has been cited in support of two different hypotheses. In the first hypothesis, cardiac autonomic dysregulation is thought to directly result from depressive and anxiety disorders and to be part of the explanation for the comorbidity of depression and anxiety with cardiovascular disease (CVD) because poor cardiac autonomic regulation constitutes a risk factor for CVD (Carney, Freedland, Miller, & Jaffe, 2002; Palatini & Julius, 2004; Tsuji et al., 1996). According to this hypothesis, psychological distress accompanying depressive and anxious mood states leads to neuroendocrine or central autonomic outflow alterations, affecting autonomic activity in the periphery (Musselman, Evans, & Nemeroff, 1998). The second hypothesis reverses the causal mechanism underlying the association. It sees cardiac autonomic dysregulation, possibly occurring in the wake of chronic stress, as a cause of allostatic adaptations in the brain and neuroendocrine systems, which in turn lead to mental health disorders (Jandackova, Britton, Malik, & Steptoe, 2016).

We voice concerns about both causal hypotheses based on three grounds. First, close inspection of the results of various correlational studies reveals them to be conflicting, as illustrated by findings of Kemp et al. (2010), Kemp et al. (2012), and Kemp et al. (2014). In 2010, these authors conducted a meta‐analysis concluding that HRV was reduced in patients with depression (Kemp et al., 2010), and in 2012 they conducted a case‐control study showing that the greatest reduction in HRV was displayed in depressed participants with comorbid generalized anxiety disorder (GAD; Kemp et al., 2012). A large cohort study in 2014 reached the contradictory conclusion that only GAD with small effect sizes and not depression or comorbid depression and anxiety disorder were associated with decreased HRV (Kemp et al., 2014). Other large studies have suggested a lack of a direct relationship between anxiety and depression with cardiac autonomic activity (Hu, Lamers, Penninx, & De Geus, 2017; Licht et al., 2015; O'Regan, Kenny, Cronin, Finucane, & Kearney, 2015). One of the explanations for these inconsistent findings might be incomplete control of confounding factors.

One particular confounding factor provides our second ground for concern in interpreting the relationship of depression and anxiety with cardiac autonomic activity as causal. Several studies, including our own, have indicated that the relationship between cardiac autonomic activity and mental health might be largely attributable to antidepressant use (Davidson, Watkins, & Owens, 2005; Hu et al., 2017; Licht, De Geus, van Dyck, & Penninx, 2010; Licht, Penninx, & De Geus, 2012; Noordam et al., 2015; O'Regan et al., 2015; Udupa, Thirthalli, & Sathyaprabha, 2011). The evidence for an impact of antidepressant use on HR and HRV is much more robust than that for depressive and anxiety disorders. Even so, ambiguity also remains in this area. Most studies found large and robust associations between the use of tricyclic antidepressants (TCAs) and cardiac autonomic dysregulation (Alvares et al., 2016; Kemp et al., 2010; Tegegne, Man, van Roon, Riese, & Snieder, 2018; Udupa et al., 2011; Van Zyl, Hasegawa, & Nagata, 2008). However, findings concerning other types of antidepressants are less consistent. For instance, selective serotonin reuptake inhibitors (SSRIs) were sometimes found to have no impact on HRV (Kemp et al., 2010; Udupa et al., 2011), a negative impact on HRV (Kemp et al., 2016; Licht et al., 2010; O'Regan et al., 2015), or a beneficial effect on HRV (Van Zyl et al., 2008) or HR (Licht et al., 2010; Van Zyl et al., 2008).

A third concern about causal hypotheses on the relationship of depression and anxiety with cardiac autonomic dysregulation is the possibility of pleiotropic genetic effects. Overlapping genetic effects may lead to an association between different biological systems that independently affect different outcomes (e.g., emotion regulation by the brain and cardiac regulation by the autonomic nervous system) without the need for a direct causal effect of one on the other. Previous studies have already demonstrated a significant genetic contribution to both internalizing psychopathology like anxiety and depression (Demirkan et al., 2011; Lubke et al., 2012; Sullivan, Neale, & Kendler, 2000) as well as resting HR (Dalageorgou et al., 2008; Russell, Law, Sholinsky, & Fabsitz, 1998) and HRV (De Geus, Van Lien, Neijts, & Willemsen, 1996) Furthermore, for many complex traits genetic pleiotropy appears to be quite common (Pickrell et al., 2016).

In short, it remains to be established whether previously found relationships between depression/anxiety and cardiac autonomic activity result from a causal effect in one or both directions, and/or may be attributable to confounding factors. Adjudicating between the above possibilities is important, as it would lead to important information for treatment strategies. For instance, if depression/anxiety directly causes cardiac autonomic dysregulation, prevention and early treatment of poor mental health might intervene in the chain of causation toward cardiac morbidity and mortality. However, if antidepressants account for the relationship between depression/anxiety and cardiac autonomic dysregulation, clinicians should exert extra caution in prescribing these, and other therapy options should be explored that render less adverse side effects. Alternatively, if shared genetic effects underlie this relationship, poor mental health and cardiac autonomic dysregulation should be treated independently, as neither causes the other.

In the present study, we used genotype and phenotype data from the large longitudinal cohort of the Netherlands Study of Depression and Anxiety (NESDA; no. of observations = 6,994), one of the largest and longest follow‐up (FU) studies allowing to examine the relationship of depressive and anxiety disorders and antidepressant use with cardiac autonomic activity. These data were combined with results from large genome‐wide association studies (GWAS) to compute polygenic risk scores (PRS) for depression (Ripke et al., 2013), resting HR (Den Hoed et al., 2013), and the root mean square of differences between successive interbeat intervals (RMSSD, a frequently used measure of HRV; Nolte et al., 2017).

Based on these data we investigated whether (a) depression/anxiety was associated with cardiac autonomic activity across four waves of assessment during a 9‐year FU period, (b) the use of three classes of antidepressants (TCAs, SSRIs, and selective serotonin and noradrenalin reuptake inhibitors [SNRIs]) was associated with cardiac autonomic activity, (c) depression/anxiety was associated with cardiac autonomic activity, independently from this effect of antidepressant use, and (d) a genetic correlation exists between depression/anxiety and cardiac autonomic activity indicating potential genetic confounding. As we expected antidepressants to impact on cardiac autonomic activity, we additionally investigated whether (e) a genetic vulnerability for cardiac autonomic activity might enhance the effect of antidepressant use on cardiac autonomic dysregulation. Such pharmacogenetic moderation might explain the discrepant findings in the literature regarding the use of some types of antidepressants, such as SSRIs. Moreover, knowledge about such a moderation effect would be clinically relevant as mental health practitioners would be advised against prescribing antidepressants especially to patients with a genetic vulnerability for cardiac autonomic dysregulation.

## 2 MATERIALS AND METHODS

### 2.1 Subjects

Subjects were participants in NESDA, a cohort study examining the long‐term course of depression and anxiety, including 2,981 participants aged 18–65 years recruited from the community, primary care, and mental health care in the Netherlands. The NESDA sample consists of persons with a current diagnosis of depression and/or anxiety disorder, a prior history of these disorders, and healthy controls. A 4‐hr baseline measurement was conducted between September 2004 and February 2007, and FU assessments took place after 2, 4, 6, 8, and 9 years. A detailed description of the rationale, objectives, and methods of the NESDA study can be found elsewhere (Penninx et al., 2008). The study protocol was approved by the ethical review board of each participating center and written informed consent was provided by all participants. The study was performed in compliance with the declaration of Helsinki.

**Figure 1:**
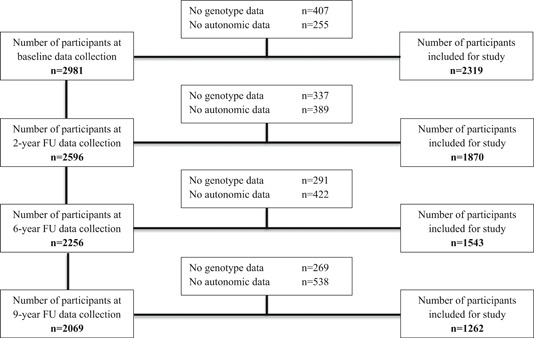
NESDA wave flow diagram. FU, follow‐up; NESDA, Netherlands Study of Depression and Anxiety

Data for the present study were drawn from baseline (*n* = 2,981), 2‐year (*n* = 2,596), 6‐year (*n* = 2,256), and 9‐year (*n* = 2,069) FU. Subjects were included if they had genetic and cardiac autonomic data. This resulted in a total of 2,319 subjects at baseline, 1,870 subjects at 2‐year FU, 1,543 subjects at 6‐year FU, and 1,262 subjects at 9‐year FU (Figure 1). Missing genetic data were mostly due to refusal to partake in DNA sampling with a smaller part lost to genotyping errors. Missing physiological data at each wave was due to the interview being held by telephone or at‐home interviews without ANS recording, equipment failure during the assessment, or poor electrocardiogram (ECG) quality.

### 2.2 Depressive/anxiety disorder

Participants were considered to have a current depressive and/or anxiety disorder if they had in the 6‐month preceding the assessment a diagnosis of major depressive disorder and/or anxiety disorder (panic disorder, social phobia, and/or GAD) according to the DSM‐IV‐based Composite International Diagnostic Interview, version 2.1 (Wittchen, 1994).

### 2.3 Antidepressant use

Participants were requested to bring their medication containers to the assessments so that medication use could be determined. Persons were considered to be using antidepressants if they reported to have used medication frequently (daily or more than 50% of the time) in the past month. We established the use of TCAs (ATC code N06AA), SNRIs (ATC code N06AX), and SSRIs (ATC code N06AB).

### 2.4 Physiological measurements

Physiological data were recorded with the “Vrije Universiteit Ambulatory Monitoring System” (VU‐AMS), an unobtrusive portable device. This device contains a six‐electrode configuration that measures ECGs and changes in thorax impedance (ICG; De Geus & Van Doornen, 1996). Data cleaning was performed with VU‐AMS software (version 4.0; VU University Amsterdam, http://www.vu-ams.nl). Movement registration through vertical accelerometry was used to remove periods where the subjects were not stationary. Bad ECG signal fragments (artifacts) were automatically detected, after which a modified version of the algorithm by Christov (Christov, 2004) was used to detect R‐wave peaks. Visual data cleaning assured that suspicious interbeat interval (IBIs) and breathing cycles were corrected or discarded when displaying irregularities.

HR and RMSSD were directly derived from the IBI time series from the ECG signal (De Geus & Van Doornen, 1996). Respiratory sinus arrhythmia (RSA), another frequently used measure of HRV, combined ECG with the respiration signal obtained from ICG, and was obtained by subtracting the shortest IBI during HR acceleration at inspiration from the longest IBI during HR deceleration at expiration for all breaths (De Geus, Willemsen, Klaver, & Van Doornen, 1995). An event marker was used to divide the assessment into different conditions. At each wave, an average score of HR, RMSSD, and RSA was made by combining the conditions that were present at all waves: a supine rest condition with blood pressure measurement (±11 min) and three sitting conditions: a psychiatric interview (±42 min), a general interview (±36 min), and a computer task (±12 min), resulting in an average total recording duration of ±107 min.

### 2.5 GWAS data and PRSs

GWAS data for HR and RMSSD were derived from Den Hoed et al. (2013), *n* = 85,787 and Nolte et al. (2017), *n* = 26,78, respectively. GWAS data for the major depressive disorder were derived from the GWAS summary statistics publicly released by the Psychiatric GWAS Consortium, excluding the dataset from 23andMe (*n* = 173,005; Ripke et al., 2013). PRSs for depression, HR, and RMSSD were based on the GWAS summary statistics (supplementary methods). As the genetic correlation between depression and anxiety has been shown to be very high (rg = 0.80; Wray et al., 2018), we have performed the analyses only with the depression‐PRS under the assumption that the results would be very similar for the anxiety‐PRS.

### 2.6 Statistical analyses

Data were analyzed using SPSS, version 22.0. Missing data were handled via full information maximum likelihood. RMSSD and RSA values were highly skewed and therefore ln‐transformed for analyses. All PRSs were standardized (mean of 0 and standard deviation [*SD*] of 1) to aid the interpretability of the results.

Generalized estimating equations (GEE) analyses were performed with an independent correlation structure to take into account within‐person correlations due to multiple observations per participant. GEE analyses were used to test the validity of HR/RMSSD‐PRS and depression‐PRS in predicting cardiac autonomic variables and depression, respectively within the NESDA sample. GEE analyses were also used to investigate whether current depression/anxiety and the use of antidepressants were associated with cardiac autonomic activity across all waves within the NESDA sample. To investigate the consistency of these relationships across waves, wave‐interaction terms were added to the model. In the case of a consistent relationship, the interaction terms are expected to equal zero. Pharmacogenetic moderation was investigated by adding HR/RMSSD‐PRS‐by‐antidepressant interaction terms to the model. Given our sample, power simulations carried out in the R “MASS” package showed that we had sufficient power (80%) to detect a pharmacogenetics moderation effect of R^2^ = 0.12 at *α* = .01 (supplementary methods). Correction for multiple testing was based on Matrix Spectral Decomposition suggested by Nyholt (2004), which corrects the α level, taking into account the correlations among the predictors (HR‐PRS, RMSSD‐PRS, current depression/anxiety, TCA use, SNRI use, and SSRI use). Accordingly, the criterion for statistical significance was set at *α* = .0084.

To test for genetic pleiotropy, we used PRS and GWAS summary‐level data to establish whether there was a genetic correlation of depression/anxiety with cardiac autonomic activity. Two types of analyses were performed. First, LD score regression (LDSC) analyses were performed on GWAS summary level data of depression and the cardiac autonomic traits to estimate their genetic covariance captured by all genotyped single‐nucleotide polymorphism (SNPs; Bulik‐Sullivan et al., 2015). Second, regression analyses established whether HR‐PRS or RMSSD‐PRS were associated with depression and anxiety and whether depression‐PRS was associated with the cardiac autonomic variables.

All analyses were adjusted for sex, age, and wave. Analyses with PRS were also adjusted for three ancestry‐informative principal components, and interaction analyses were additionally adjusted for covariate‐by‐gene and covariate‐by‐exposure interaction terms, as suggested by Keller (2014).

## 3 RESULTS

Table 1 shows the characteristics of our sample at baseline (*n* = 2,319), 2‐year FU (*n* = 1,870), 6‐year FU (*n* = 1,543), and 9‐year FU (*n* = 1,262). For instance, at baseline, our sample had a mean age of 42.4 years (*SD* = 13.0) and included 66.1% females.

**Table 1:** Sample characteristics

	Wave			
	Baseline (*n* = 2319)	2‐Year FU (*n* = 1870)	6‐Year FU (*n* = 1543)	9‐Year FU (*n* = 1262)
Sociodemographic				
*Age, years (mean ± *SD*)	42.4 ± 13.0	44.6 ± 13.1	48.6 ± 13.0	51.2 ± 13.1
*Female (%)	66.1	65.9	65.3	63.5
Mental health				
*Current depression/anxiety (%)	82.1	38.3	28.3	27.6
*Depression (%)	47.7	59.5	57.4	54.6
*Generalized anxiety disorder (%)	7.4	20.5	20.0	8.4
*Social phobia (%)	29.1	37.5	29.7	32.4
*Panic disorder (%)	11.6	30.5	28.8	17.3
*IDS‐SR score (mean ± *SD*)	24.9 ± 13.3	25.0 ± 12.1	26.2 ± 12.3	25.1 ± 12.4
*BAI score (mean ± *SD*)	13.9 ± 10.5	14.4 ± 9.6	14.8 ± 10.7	14.3 ± 9.8
Antidepressant use (%)				
*Use of TCA	2.8	3.0	3.3	4.0
*Use of SNRI	4.4	4.4	4.0	4.0
*Use of SSRI	17.6	15.1	12.5	12.4
Cardiac autonomic variables				
*HR, beat/min (mean ± *SD*)	71.9 ± 9.6	72.7 ± 9.7	71.6 ± 9.6	70.7 ± 9.8
*RMSSD, ms (median [IQR])	31.6 (22.3–45.5)	30.0 (20.5–43.9)	22.2 (15.7–32.1)	27.0 (19.0–38.6)
*RSA, ms (median [IQR])	38.0 (26.5–53.9)	36.6 (24.9–52.3)	38.9 (26.4–55.6)	44.6 (31.6–62.0)

Abbreviations: BAI, beck anxiety inventory; FU, follow‐up; HR, heart rate; IDS‐SR, inventory of depressive symptomatology‐self report; IQR, interquartile range; RMSSD, root mean square of differences between successive interbeat intervals; RSA, respiratory sinus arrhythmia; *SD*, standard deviation; SNRI, selective serotonergic and noradrenergic reuptake inhibitors; SSRI, selective serotonin reuptake inhibitors; TCA, tricyclic antidepressant.

### 3.1 The association of depression/anxiety and antidepressant use with cardiac autonomic activity

Table 2 shows the results of the GEE analyses establishing the relationship of depression/anxiety and antidepressant use with the cardiac autonomic activity. Without adjustment for antidepressant use, depression/anxiety was not significantly associated with HR (B = −0.228; *p* = .50), but (marginally) significantly associated with RMSSD (B = −0.047; *p* = .011) and RSA (B = −0.052; *p* = .001) across waves. However, after adjustment for antidepressant use, all associations between depression/anxiety and cardiac autonomic variables rendered nonsignificantly. In addition, no significant wave‐interaction effects were found, suggesting that the associations did not differ across waves (not tabulated).

**Table 2:** Main and interaction effects of PRS and current depression/anxiety/antidepressant use on the cardiac autonomic trait (no. of observations = 6,994)

	Cardiac autonomic trait								
	HR			lnRMSSD			lnRSA		
	*B*	*p*	*R* ^2^	*B*	*p*	*R* ^2^	*B*	*p*	*R* ^2^
Main effects									
HR‐PRS	**1.810**	**<.001**	.063	**−0.067**	**<.001**	.129	**−0.046**	**<.001**	.284
RMSSD‐PRS	**−0.755**	**<.001**	.033	**0.061**	**<.001**	.125	**0.049**	**<.001**	.284
Current depression/anxiety	−0.228	.50	.028	−0.047	.011	.119	**−0.052**	**.001**	.280
Current depression/anxiety	−0.593	.070	.068	0.010	.58	.169	0.007	.67	.333
TCA use	**10.292**	**<.001**		**−0.651**	**<.001**		**−0.652**	**<.001**	
SNRI use	**3.171**	**<.001**		**−0.312**	**<.001**		**−0.307**	**<.001**	
SSRI use	**−1.134**	**.006**		**−0.134**	**<.001**		**−0.150**	**<.001**	
Pharmacogenetic moderation									
HR‐PRS*TCA use	1.086	.31	.108	−0.104	.070	.188	−0.085	.10	.344
HR‐PRS*SNRI use	0.809	.17		0.008	.84		0.033	.34	
HR‐PRS*SSRI use	0.310	.40		−0.011	.61		−0.013	.50	
RMSSD‐PRS*TCA use	2.281	.029	.079	0.070	.20	.185	0.045	.37	.345
RMSSD‐PRS*SNRI use	0.176	.81		−0.048	.17		−0.063	.054	
RMSSD‐PRS*SSRI use	0.488	.26		−0.005	.83		−0.003	.87	

*Note:* GEE analyses were adjusted for sex, age, and wave. Analyses with PRS were also adjusted for ancestry‐informative principal components. Analyses with PRS‐interaction terms were additionally adjusted for covariate‐by‐gene and covariate‐by‐exposure interaction terms. R2 values are based on regression analyses. Boldface indicates statistical significance (*p* < .0084).

Abbreviations: HR, heart rate; PRS, polygenic risk scores; RMSSD,  root mean square of differences between successive interbeat intervals; RSA, respiratory sinus arrhythmia; SNRI, selective serotonergic and noradrenergic reuptake inhibitors; SSRI, selective serotonin reuptake inhibitors; TCA, tricyclic antidepressant.

* interaction term

TCA (*B* = 10.292; *p* < .001) and SNRI (*B* = 3.171; *p* < .001) use were associated with a significantly higher HR, while SSRI use was associated with a lower HR (*B* = −1.134; *p* = .006) across waves. Use of all antidepressants was associated with lower RMSSD and RSA, with the largest effect of TCA (RMSSD: *B* = −0.651; *p* < .001; RSA: *B* = −0.652; *p* < .001), followed by SNRI (RMSSD: *B* = −0.312; *p* < .001; RSA: *B* = −0.307; *p* < .001) and SSRI (RMSSD: *B* = −0.134; *p* < .001, RSA: *B* = −0.150; *p* < .001). No significant wave‐interaction effects were found, suggesting that the associations were consistent across waves (not tabulated).

### 3.2 The genetic association of depression/anxiety with cardiac autonomic activity

First, we tested the validity of HR/RMSSD‐PRS in predicting cardiac autonomic variables within NESDA, and the validity of the depression‐PRS in predicting depression diagnosis within NESDA. Figure S1 shows the explained variances in cardiac autonomic traits by HR‐PRS and RMSSD‐PRS per wave for the various PRS constructed in different ways. These findings support the consistency of the contribution of polygenetic risk factors to variance in cardiac autonomic activity across 9‐year FU data. We pursued the analyses with the best performing PRS for HR and RMSSD (i.e., LDpred [0.03], which assumes that 3% of all variants are causal for the trait of interest). As expected, GEE analyses showed that HR‐PRS was positively associated with HR (*B* = 1.810; *p* < .001), and negatively associated with RMSSD (*B* = −0.067; *p* < .001) and RSA (*B* = −0.046; *p* < .001) across all waves in NESDA. Also in line with expectations, RMSSD‐PRS was negatively associated with HR (*B* = −0.755; *p* < .001), and positively associated with RMSSD (*B* = 0.061; *p* < .001) and RSA (*B* = 0.049; *p* < .001) across all waves (Table 2). In addition, depression‐PRS was significantly associated with depression diagnosis across all waves (*B* = 0.186; *p* < .001**;** Figure S2).

After confirming the validity of HR/RMSSD‐PRS and depression‐PRS, we investigated the genetic correlation between depression/anxiety and cardiac autonomic activity. LDSC analyses using GWAS summary statistics yielded small and statistically nonsignificant genetic correlation estimates of depression with HR (rg = 0.08; *p* = .062) and RMSSD (rg = 0.0755; *p* = .29). Congruently, we found no associations of HR‐PRS (*B* = 0.017; *p* = .53) and RMSSD‐PRS (*B* = 0.005; *p* = .84) with depression/anxiety in the NESDA dataset (Figure S3). We also found depression‐PRS not to be associated with HR (*B* = 0.301; *p* = .092), RMSSD (*B* = −0.015; *p* = .14), or RSA (*B* = −0.014; *p* = .094). As these relationships might be affected by antidepressant use, we reran the analyses with additional adjustment for use of TCA, SNRI, and SSRI, but the results remained insignificant. The LDSC and PRS analyses suggest that genetic pleiotropic effects do not, or only very weakly, explain the relationship between these traits.

### 3.3 Pharmacogenetic moderation

The interaction terms testing pharmacogenetic moderation of the HR/RMSSD‐PRS effect on the relationship between antidepressant use and cardiac autonomic variables were all nonsignificant.

### 3.4 Posthoc analyses with depression/anxiety severity scores and antidepressant derived daily dose

To check for possible dose–response relationships of depression/anxiety and antidepressant use with cardiac autonomic activity, we reran the GEE analyses with depression/anxiety severity scores and antidepressant derived daily dose (DDD; Table S1). Similar to the results regarding depression/anxiety diagnosis, all associations between depression/anxiety severity and cardiac autonomic variables rendered nonsignificant after adjustment for antidepressant use. Higher TCA DDD was significantly associated with higher HR (*B* = 2.218; *p* = .007), lower RMSSD (*B* = −0.249; *p* < .001), and RSA (*B* = −0.195; *p* < .001). In addition, higher SNRI DDD was significantly associated with lower RMSSD (*B* = −0.101; *p* < .001), and higher SSRI DDD was significantly associated with lower RMSSD (*B* = −0.067; *p* < .001) and RSA (*B* = −0.060; *p* = .001).

### 3.5 Posthoc analyses using IBI‐adjusted HRV variables

Because the ability of RMSSD and RSA to index cardiac autonomic activity may be associated with the prevailing HR, we reran the GEE analyses with IBI, and HRV variables corrected for the mean IBI (the reciprocal of HR) as suggested by De Geus, Gianaros, Brindle, Jennings, and Berntson (2019; Table S2). Overall, these results were similar to the original GEE analyses, suggesting that the present findings were not merely due to individual differences in HR.

## 4 DISCUSSION

It is unclear whether the previously found relationship of depressive and anxiety disorders with cardiac autonomic activity results from a causal effect in either direction or may be attributable to confounding factors. We investigated possible confounding by genetic pleiotropic effects in the relationship between depression and anxiety and cardiac autonomic activity but did not find evidence for this. The current study indicates that the above relationship does not result from a causal pathway or genetic pleiotropy, but is driven by the use of antidepressants. The use of TCAs, SNRIs, and SSRIs was consistently associated with cardiac autonomic dysregulation across the 9‐year FU.

Previous NESDA studies indicated that there was little evidence for a direct association of depression and anxiety with cardiac autonomic dysregulation (Hu et al., 2017; Licht et al., 2010). This was confirmed by the current 9‐year FU study. In addition, no genetic correlation between these domains was found: LDSC analyses showed no significant association between GWAS summary level data of depression and cardiac autonomic activity. In keeping, neither HR‐PRS nor RMSSD‐PRS predicted diagnosed depression and anxiety, and the depression‐PRS did not predict these cardiac autonomic variables. The finding that a genetic correlation is lacking between these traits is relevant, as this suggests that depression and anxiety are not inevitably linked with cardiac autonomic dysregulation, but that interventions are possible to break this deleterious relationship.

The NESDA results align with other large cohort studies showing that depression and anxiety may not directly cause cardiac autonomic dysregulation, but these effects are, at large or fully, attributable to the use of antidepressants (Noordam et al., 2015; O'Regan et al., 2015; Tegegne et al., 2018). We found the use of antidepressants to be consistently associated with cardiac autonomic activity across 9‐year longitudinal data. In line with our previous studies across shorter time spans (Hu et al., 2017; Licht et al., 2010; Licht et al., 2012), we found a detrimental effect on the cardiac autonomic activity of TCA use, followed by SNRI and SSRI use. SSRI use might even have a slightly beneficial effect on HR. Additional analyses with antidepressant DDD indicated that there might be a dose–response effect of the use of TCAs, SNRIs, and SSRIs on cardiac autonomic dysregulation. Although these effects are robustly found within the literature, not all studies agree on the effects of certain types of antidepressants, especially SSRIs. We, therefore, hypothesized that the association between antidepressant use and cardiac autonomic dysregulation might be affected by pharmacogenetic moderation. However, we did not find a moderation effect of genetic risk for high HR or low HRV, meaning that the effects of antidepressant use were not amplified by the genetic vulnerability, or ameliorated by genetic resilience. Nonetheless, this study adds to the increasing evidence that almost all antidepressants affect cardiac autonomic activity (Kemp et al., 2014; O'Regan et al., 2015), with the strongest detrimental effects associated with TCAs, and the mildest effects with SSRIs. These results are in correspondence with recent findings in a very large general population sample (*n* = 149,205), where similar effects on HRV were found for TCAs, SNRIs, and SSRIs (Tegegne et al., 2018). Although the relevant mechanisms are not entirely understood, it is thought that antidepressants influence relay nuclei of the parasympathetic nervous system in the brain stem (Raul, 2003), inhibit cardiac vagal tone by exerting anticholinergic activity (Lavretsky, Lesser, Wohl, & Miller, 1998), and/or inhibit the reuptake of norepinephrine in the heart (Esler, Hasking, Willett, Leonard, & Jennings, 1985). In addition, the electrophysiological effects that antidepressants exert on ion channels (e.g., the blockage of sodium and/or potassium channels) may affect the cardiac action potential, thereby causing prolongation of the QT interval and other cardiovascular side effects (Nachimuthu, Assar, & Schussler, 2012; Sala et al., 2000). For instance, TCAs are thought to affect a broad range of receptor pathways, including a significant inhibition of central cholinergic neurotransmission, impaired neuronal uptake of norepinephrine, and the blockage of sodium channel conductance. Likewise, SNRIs are suggested to stimulate cardiac sympathetic activity by increasing peripheral norepinephrine concentrations and blocking cardiac sodium channel conductance (Waring, 2012).

The major strength of this study is that it is one of the largest studies with the longest FU to investigate the relationship of diagnosed depression and anxiety and different classes of antidepressants with cardiac autonomic activity, and the first to address the role of genetics. A general limitation of the use of the PRS is the limited amount of complex trait variance explained. In the present analyses, PRS for autonomic traits captured 0.4–4.2% of the related trait variance. The explanatory power of PRS is a function of the discovery GWAS sample size: a larger training dataset allows smaller sampling variance on the individual SNP effect estimates, leading to a more powerful PRS. For our study, we used the largest GWAS available up to date. In the future, PRS derived from larger GWAS could be used to further confirm the present findings. The question of adequate power is particularly relevant with regard to the pharmacogenetic moderation analyses. Our sample (no. of observations = 6,994) had sufficient power (80%) to detect a gene‐by‐antidepressant interaction effect of R2 = 0.12 for antidepressant use at *α* = .01. Therefore, our analyses cannot exclude interaction effects smaller than these. However, an effect of smaller size might not be of clinical relevance and probably could not explain the discrepant findings in this research area. Another limitation is that the used discovery (and target) sample mainly includes individuals of European descent. This lack of ancestral diversity limits the generalizability of our findings. This study focused on diagnosed depression/anxiety and symptom severity. However, the association of specific symptoms or symptom clusters of depression and anxiety on autonomic dysregulation cannot be ruled out by our findings. In addition, we have not investigated the effect of the course of depression/anxiety, such as chronicity, on cardiac autonomic activity. However, previous NESDA research over 6 years has shown that neither chronic depression/anxiety nor onset or remission of depression/anxiety was associated with the change in cardiac autonomic functioning (Hu et al., 2017). Therefore, we do not expect disease course to influence our findings. Lastly, although the found association between antidepressant DDD and cardiac autonomic dysregulation suggests a dose–response effect, the current study did not include many participants who used antidepressants in high dosage (DDD was equal or less than 1.5 for over 95% of the persons using antidepressants). Future studies might further explore the effects of antidepressant dosage, course of the disease, as well as different depressive and anxious symptom dimensions on cardiac autonomic activity. This study is unique in addressing the role of genetic pleiotropy and pharmacogenetic moderation in the relationship between depression, anxiety, antidepressant use, and cardiac autonomic dysregulation across four waves in 9 years. This strength also means that future replication studies in independent samples are needed to verify or falsify the current findings regarding genetic correlation and moderation.

## 5 CONCLUSION

To conclude, in this large longitudinal dataset, we found no evidence for a causal relationship between depressive and anxiety disorders with cardiac autonomic activity. We found robust effects of antidepressant use, with the strongest detrimental effect of TCAs, followed by SNRIs and SSRIs. These results suggest that previously reported associations of depression and anxiety with cardiac autonomic dysregulation are likely caused by the confounding of antidepressant use. The finding that it is not genetic pleiotropy, but rather antidepressant use that confounds the relationship of depression and anxiety with cardiac autonomic activity is important, as it implies we are able to intervene in this harmful relationship. For instance, clinicians should take further caution when prescribing certain classes of antidepressants, especially TCAs and SNRIs, and more so when treating patients with poor cardiovascular health.

## CONFLICT OF INTERESTS

The authors declare that there are no conflict of interests.

## Supporting information

Supplementary information

## Data Availability

The data that support the findings of this study are available on request from the corresponding author. The data are not publicly available due to privacy or ethical restrictions.
