# Supplementary material for: The association of depression and anxiety with cardiac autonomic activity: The role of confounding effects of antidepressants
Source: Depress Anxiety. 2019 Oct 17;36(12):1163–72. doi: 10.1002/da.22966 (PMC6916630; doi:10.1002/da.22966)
Supplement: Supplementary file 1 — Supplementary information [file DA-36-1163-s001.docx]

**Supplementary Methods**

*GWAS data and PRSs*

Genotyping (95% of the samples on Affymetrix 6.0 Human SNP array and the remaining on Perlegen-Affymetrix 5.0 array), quality control steps and imputation were previously described in detail.^1^ Briefly, after platform-specific QC, in order to combine the SNPs genotyped in each platform, the two datasets were imputed using the GoNL (Genome of the Netherlands) reference panel^2^ and then merged. From the imputed dataset only the SNPs genotyped in the original platforms were retained, creating a unique cross-platform imputed dataset. After more stringent QC, the cross-platform imputed dataset included ~1.2M SNPs.

PRS for depression, HR, and RMSSD were based on the GWAS summary statistics described in the previous paragraph. ﻿Since NESDA data were part of the GWAS for depression, we re-ran the meta-analysis after removal of overlapping samples. Since NESDA data was part also of the RMSSD GWAS, the meta-analysis was corrected for the NESDA sample using MetaSubtract (https://cran.r-project.org/web/packages/MetaSubtract/index.html). Summary statistics of the discovery were lifted to human genome build hg19 (HR and RMSSD) and filtered by removing strand ambiguous variants and SNPs with MAF < 0.01. Overlapping SNPs (~1M) between the cross-platform GoNL were imputed and those retained from the discovery summary statistics were carried forward. PRS were calculated as the number of risk alleles weighted by effect sizes from the discovery statistics. Selection of SNPs and effect sizes was based on two methods. Firstly, clumping + P-thresholding method using PLINK was applied. SNPs were selected using p-value-informed LD clumping (250kb window, r^2^=0.25) using 1000 unrelated individuals randomly selected from NESDA for LD for reference. Nine sets of score alleles and related effect size were selected based on different significance thresholds (P<10x^-8^, P<10x^-7^, P<10x^-6^, P<10x^-5^, P<10x^-4^, P<10x^-3^, P<0.05, P<0.5, P<1) of the discovery samples associations. Additionally, PRSs were built using the LDpred method,^3^ which has shown improved predictive performance compared with the previous method by modeling a Bayes prior on effect sizes and including LD information. The fraction of causal SNPs was set as infinitesimal, assuming the entire genome as causal, and at 3% consistent with the best performing fraction for cardiovascular disorders previously reported.^3^

*References*

1 Mbarek H, Milaneschi Y, Hottenga J-J, Ligthart L, De Geus EJC, Ehli EA *et al.* Genome-wide significance for PCLO as a gene for major depressive disorder. *Twin Res Hum Genet* 2017; **20**: 267–270.

2 Francioli LC, Menelaou A, Pulit SL, Van Dijk F, Palamara PF, Elbers CC *et al.* Whole-genome sequence variation, population structure and demographic history of the Dutch population. *Nat Genet* 2014; **46**: 818–825.

3 Vilhjálmsson BJ, Yang J, Finucane HK, Gusev A, Lindström S, Ripke S *et al.* Modeling linkage disequilibrium increases accuracy of polygenic risk scores. *Am J Hum Genet* 2015; **97**: 576–592.

*Power simulation*

**R script**

rm(list=ls(all=TRUE))

library(MASS)

#

# model HR = b0 + b1*PGS + b2*E + b3*PGS*E + residual

# PGS standardized

# E coded 0/1

# parameter of interest b3, i.e., the interaction term

#

wr=F # write to external file

# -------------------------------------------------------------

# input

T=4 # number of repeated measures

Ntot=N=2319 # sample size

r=.5 # correlation among T HR meaures

p_exp0=.827 # proportion of non-exposed AD (E=0) =.827

R2exp0=.1 # prediction in non-exposed group

NES=49 # number of effect sizes

R2exp1s=seq(.1,.4,len=NES) # prediction in exposed group

d=.3 # differences in intercept exposed vs non-exposed

# # d does not affect the power to reject b3=0

alpha=.01 # alpha for testing the interaction

#

# there are N =Ntot cases, T= time points

# T=1 2 3 4

# probobs=c(0,0,0,1) # the simuation is: no missing values, 100% have T=4 observations

# T=1 2 3 4

probobs=c(.145,.194,.255,.406) # in the case 40.6% have 4 observation, 25.5% have 3, 19.4% have 2 and 14.5% have 1.

# should sum to 1

print(sum(probobs))

#

# ------------------------------------------------------------

#

#

p_exp1=1-p_exp0 # % E=1

b0exp0=0 # intercept in E=0 group

b0exp1=d # intercept in E=1 group

b1exp0=sqrt(R2exp0) # R2 in E=0 group

#

#

Ns=rep(0,4)

ESS=rep(0,4)

for (i in 1:4) {

t=i

Ns[i]=ESS[i]=(t*Ntot*probobs[i]) /(1+(t-1)*r) # get the effective sample size

}

#

N=sum(Ns) # effective sample

#

N0=round(N*p_exp0) # N0 number of non-exposed

N1=round(N*p_exp1) # N1 number of exposed

N=N1+N0 # total sample size

#

# simulate residual and pgs exactly

R=diag(2) # both standardized

d0=mvrnorm(N0,rep(0,2),Sigma=R,emp=T)

d1=mvrnorm(N1,rep(0,2),Sigma=R,emp=T)

#

# build data given each of the NES effectsizes

# and calculate power

#

power1=rep(0,NES)

ii=0

for (R2exp1 in R2exp1s) {

ii=ii+1

b1exp1=sqrt(R2exp1)

#

d=matrix(0,N,3)

d[1:N0,1] =b1exp0*d0[,2]+b0exp0+d0[,1] # E=0 group

d[(N0+1):N,1]=b1exp1*d1[,2]+b0exp1+d1[,1] # E=1 group

d[1:N0,2]=d0[,2] # PGS

d[1:N0,3]=0 # E

d[(N0+1):N,2]=d1[,2] # PGS

d[(N0+1):N,3]=1 # E

d=as.data.frame(d)

colnames(d)=c('hr','pgs','expo')

#

# do the analyses and get power

res_lm1=lm(hr~pgs*expo,data=d)

# based on WALD test / ASYMPTOTIC chi2 test

ncp=summary(res_lm1)$coefficients[4,3]^2

crit=qchisq(alpha,1,lower=F)

power1[ii]=pchisq(crit,1,ncp,lower=F)

#

#

if (wr) {

d=cbind(rep(ii,N),d)

write.table(d,file='d1',col.names=TRUE, row.names=FALSE,append=T)

}

}

#

x=R2exp1s-R2exp0

ti=paste('ESS=',as.character(N),' alpha=',as.character(alpha))

plot(x,power1,type='b',xlab='es',main=ti)

lines(x,rep(.8,NES),type='l',lwd=2,col=2)

x

power1

**
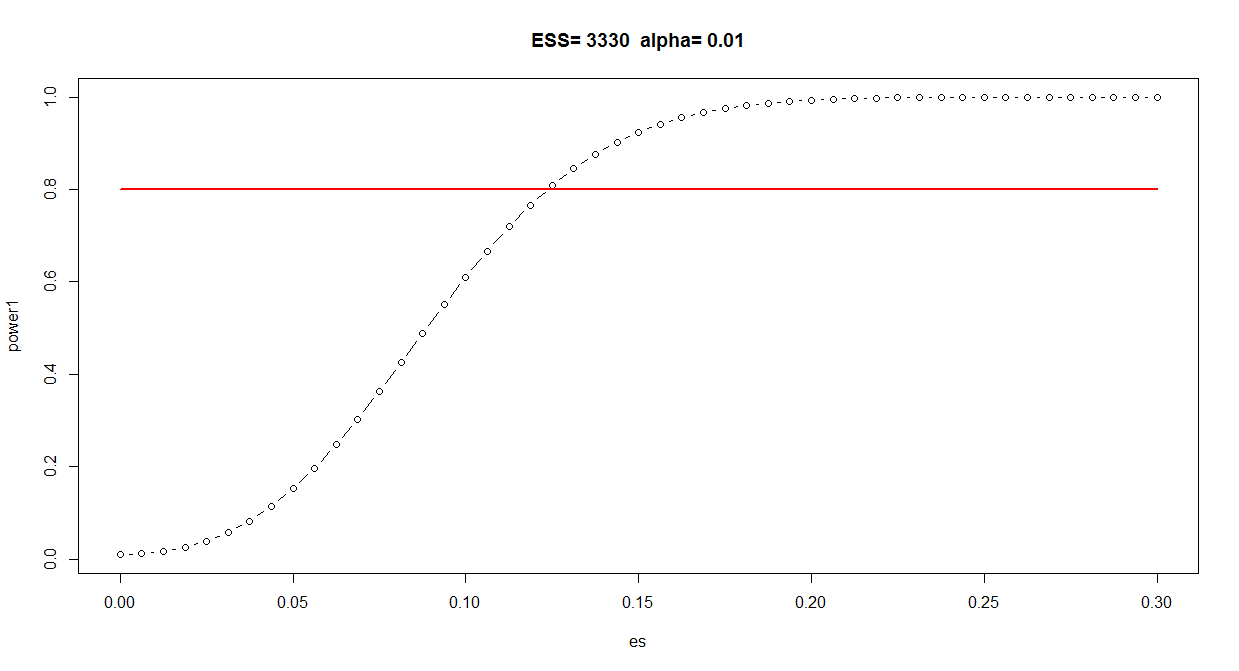
**

| **Supplementary table 1. Main and interaction effects of PRS and depression/anxiety severity and antidepressant DDD on cardiac autonomic trait (no. observations = 6994)** | | | | | | | | |
| --- | --- | --- | --- | --- | --- | --- | --- | --- |
|  | Cardiac autonomic trait | | | | | | | |
|  | HR | |  | lnRMSSD | |  | lnRSA | |
| **Main effects** | B | p |  | B | p |  | B | p |
| IDS-SR score | 0.013 | .34 |  | **-0.003** | **<.001** |  | **-0.003** | **<.001** |
| BAI score | 0.018 | .29 |  | **-0.003** | **.001** |  | **-0.004** | **<.001** |
| TCA DDD (n=224)^a^ | **2.218** | **.007** |  | **-0.249** | **<.001** |  | **-0.195** | **<.001** |
| SNRI DDD (n=296)^a^ | 0.822 | 0.14 |  | **-0.101** | **<.001** |  | -0.082 | .011 |
| SSRI DDD (n=1041)^a^ | 0.507 | .14 |  | **-0.067** | **<.001** |  | **-0.060** | **.001** |
| IDS-SR score | -0.014 | .49 |  | -0.001 | .49 |  | -0.001 | .43 |
| BAI score | 0.020 | .42 |  | 3.2E-5 | .98 |  | 3.9E-4 | .77 |
| TCA DDD | **6.905** | **<.001** |  | **-0.477** | **<.001** |  | **-0.448** | **<.001** |
| SNRI DDD | **1.619** | **<.001** |  | **-0.166** | **<.001** |  | **-0.157** | **<.001** |
| SSRI DDD | **-0.441** | **.074** |  | **-0.080** | **<.001** |  | **-0.081** | **<.001** |
| **Pharmacogenetic moderation** |  |  |  |  |  |  |  |  |
| HR-PRS*TCA DDD | -0.001 | >.99 |  | 0.009 | .86 |  | 0.015 | .76 |
| HR-PRS*SNRI DDD | 0.113 | .80 |  | 0.017 | .45 |  | 0.043 | .054 |
| HR-PRS*SSRI DDD | 0.406 | .086 |  | -0.022 | .088 |  | -0.026 | .030 |
| RMSSD-PRS*TCA DDD | 0.616 | .46 |  | 0.095 | .048 |  | 0.105 | .021 |
| RMSSD-PRS*SNRI DDD | -0.081 | .85 |  | -0.033 | .10 |  | -0.040 | .035 |
| RMSSD-PRS*SSRI DDD | 0.176 | .51 |  | 0.013 | .40 |  | 0.016 | .29 |
| *Note:* PRS = polygenic risk scores. HR = heart rate. RMSSD = root mean square of differences between successive interbeat intervals. RSA = respiratory sinus arrhythmia. TCA = tricyclic antidepressant. SNRI = selective serotonergic and noradrenergic reuptake inhibitors. SSRI = selective serotonin reuptake inhibitors. IDS-SR = Inventory of Depressive Symptomatology-Self Report. BAI = Beck Anxiety Inventory. DDD = derived daily dose.  GEE analyses were adjusted for sex, age, and wave. Analyses with PRS were also adjusted for ancestry-informative principal components. Analyses with PRS-interaction terms were additionally adjusted for covariate-by-gene and covariate-by-exposure interaction terms.  ^a^These analyses were performed on users of the respective antidepressant (and excluded non-users) to investigate dose-response effects.  Boldface indicates statistical significance (p<.0084). | | | | | | | | |

| **Supplementary table 2. Main and interaction effects of PRS and current depression/anxiety/antidepressant use on cardiac autonomic trait (no. observations = 6994)** | | | | | | | | |
| --- | --- | --- | --- | --- | --- | --- | --- | --- |
|  | Cardiac autonomic trait | | | | | | | |
|  | IBI | |  | Ln(RMSSD/IBI) | |  | Ln(RSA/IBI) | |
| **Main effects** | B | p |  | B | p |  | B | p |
| HR-PRS | **-21.799** | **<.001** |  | **-0.041** | **<.001** |  | -0.019 | .012 |
| RMSSD-PRS | **10.692** | **<.001** |  | **0.056** | **<.001** |  | **0.044** | **<.001** |
| Current depression/anxiety | 4.306 | .10 |  | -0.019 | .12 |  | -0.014 | .18 |
| Current depression/anxiety | 6.225 | .015 |  | 0.005 | .71 |  | 0.008 | .45 |
| TCA use | **-95.067** | **<.001** |  | **-0.463** | **<.001** |  | **-0.464** | **<.001** |
| SNRI use | **-42.116** | **<.001** |  | **-0.247** | **<.001** |  | **-0.233** | **<.001** |
| SSRI use | **21.202** | **<.001** |  | **-0.119** | **<.001** |  | **-0.143** | **<.001** |
| **Pharmacogenetic moderation** |  |  |  |  |  |  |  |  |
| HR-PRS*TCA use | 2.469 | .77 |  | -0.073 | .10 |  | -0.058 | .15 |
| HR-PRS*SNRI use | 4.824 | .42 |  | 0.014 | .60 |  | 0.014 | .63 |
| HR-PRS*SSRI use | -1.329 | .74 |  | -0.003 | .85 |  | 0.001 | .93 |
| RMSSD-PRS*TCA use | -9.581 | .28 |  | 0.084 | .072 |  | 0.048 | .25 |
| RMSSD-PRS*SNRI use | -3.734 | .52 |  | -0.039 | .15 |  | -0.056 | .029 |
| RMSSD-PRS*SSRI use | -2.076 | .61 |  | -0.002 | .90 |  | -0.010 | .56 |
| *Note:* PRS = polygenic risk scores. IBI = interbeat interval. RMSSD = root mean square of differences between successive interbeat intervals. RSA = respiratory sinus arrhythmia. TCA = tricyclic antidepressant. SNRI = selective serotonergic and noradrenergic reuptake inhibitors. SSRI = selective serotonin reuptake inhibitors.  GEE analyses were adjusted for sex, age, and wave. Analyses with PRS were also adjusted for ancestry-informative principal components. Analyses with PRS-interaction terms were additionally adjusted for covariate-by-gene and covariate-by-exposure interaction terms.  Boldface indicates statistical significance (p<.0084). | | | | | | | | |


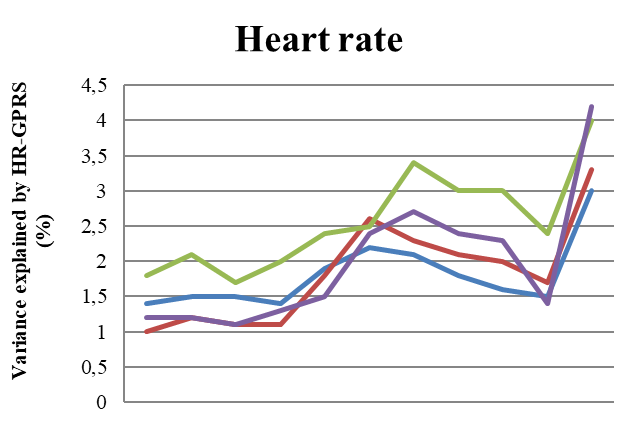

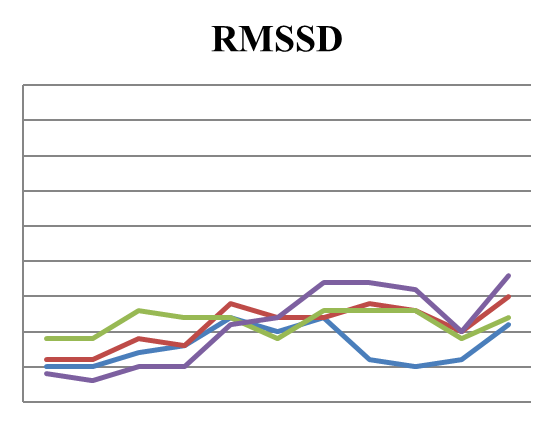

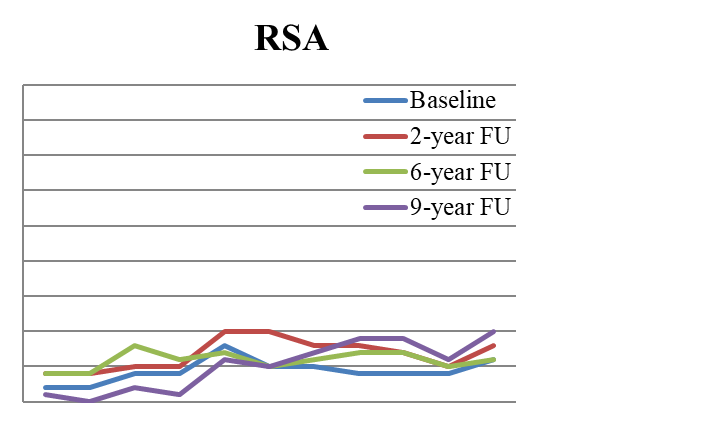

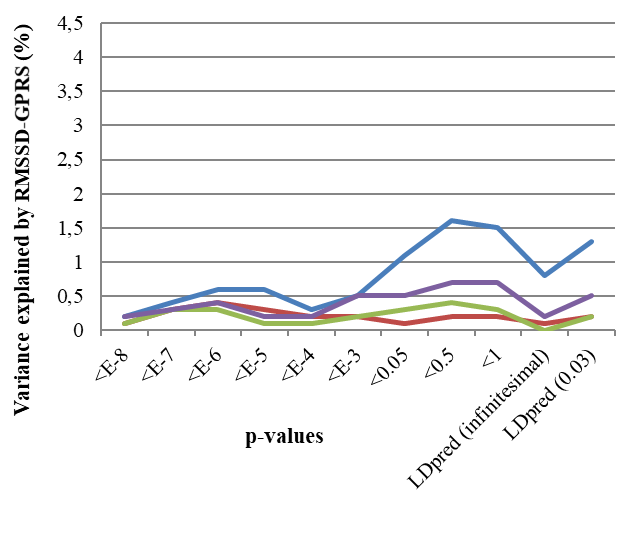

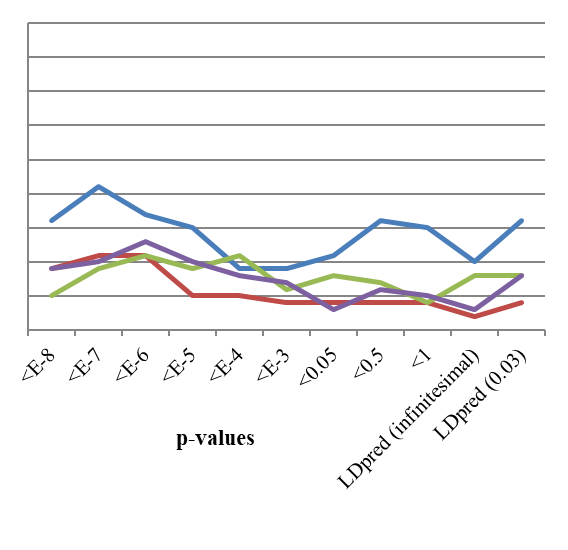

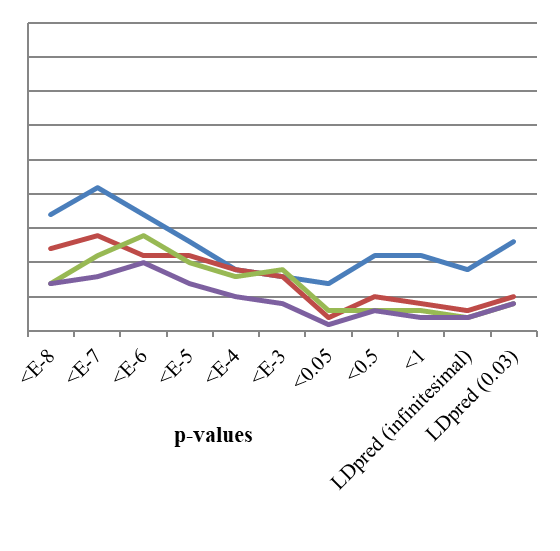


**Supplementary figure 1.** Explained variance in HR, RMSSD, and RSA traits by polygenic risk scores (PRS) for HR and RMSSD within NESDA for baseline (n=2319), 2-year FU (n=1870), 6-year FU (n=1543), and 9-year FU (n=1262). Explained variance was established using regression analyses by calculating the change in R^2^ when adding HR/RMSSD-PRS to the model.


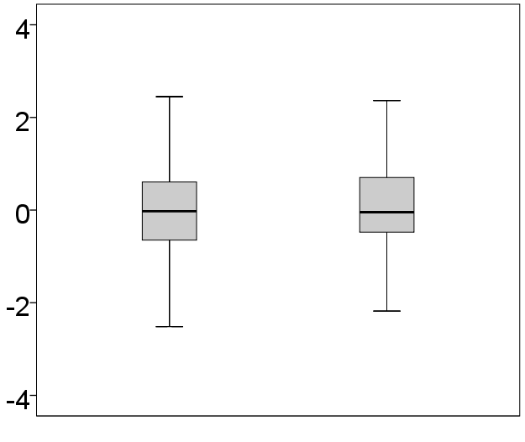

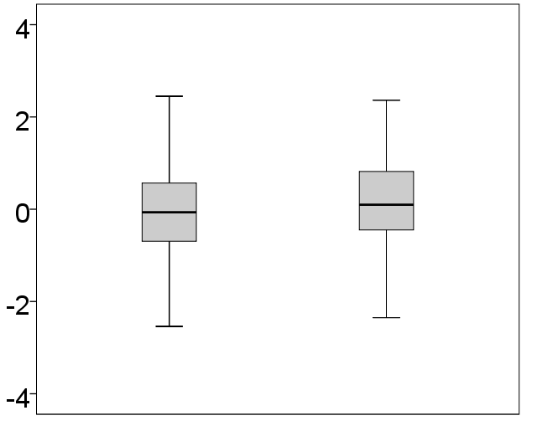

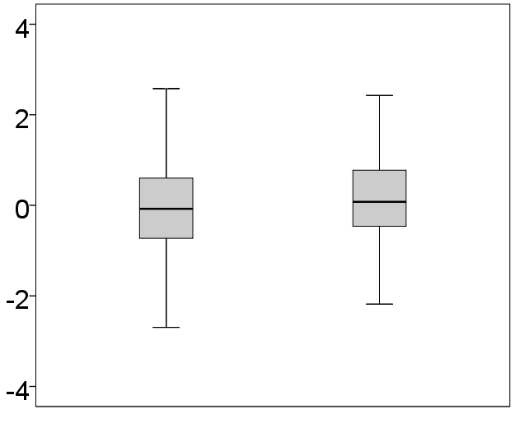


**Controls Current**

**depression/anxiety**

**Baseline**

**2-year FU**

**6-year FU**

**9-year FU**

**Depression-PRS**

***

***

**

**Supplementary figure 2.** Distribution of depression-PRS at baseline, two-, six-, and nine-year follow-up for controls and current depression/anxiety (see Table 1 for sample size per wave). ** Indicates that persons with current depression score higher on depression-PRS than controls at p<.01. *** Indicates that persons with current depression score higher on depression-PRS than controls at p<.001.


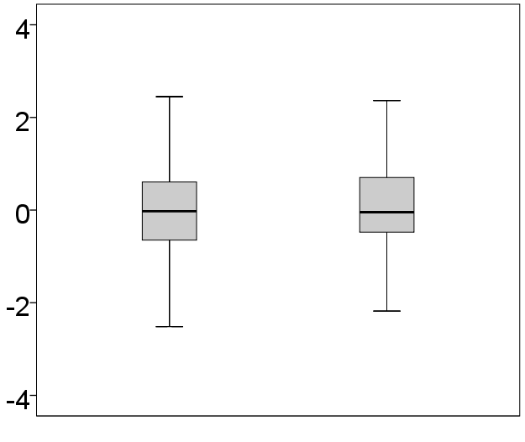


**Controls Current**

**depression/anxiety**

**Controls Current**

**depression/anxiety**

**Controls Current**

**depression/anxiety**

**Controls Current**

**depression/anxiety**

**Supplementary figure 3.** Distribution of HR-PRS and RMSSD-PRS at baseline, two-, six-, and nine-year follow-up for controls and current depression/anxiety (see Table 1 for sample size per wave).

**Baseline**

**2-year FU**

**6-year FU**

**9-year FU**


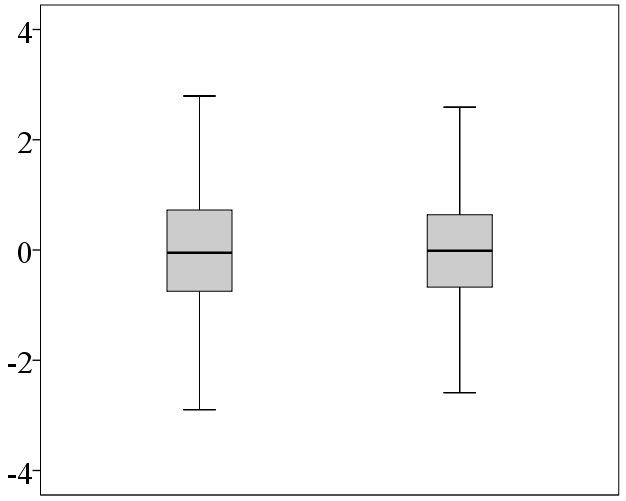

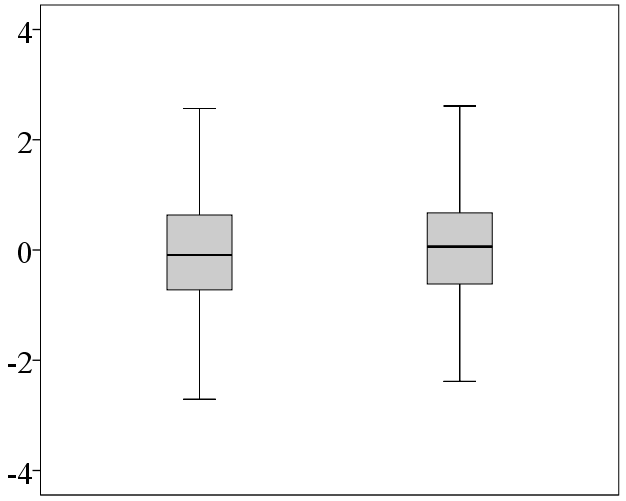

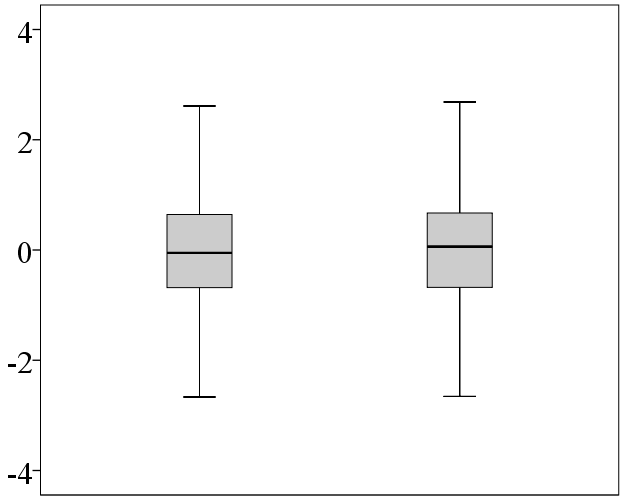

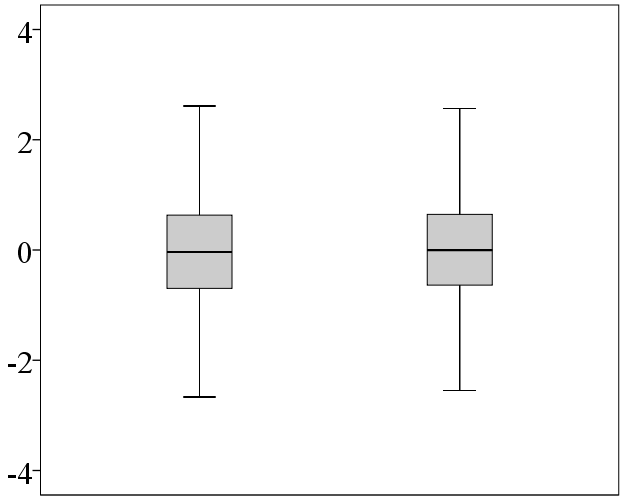

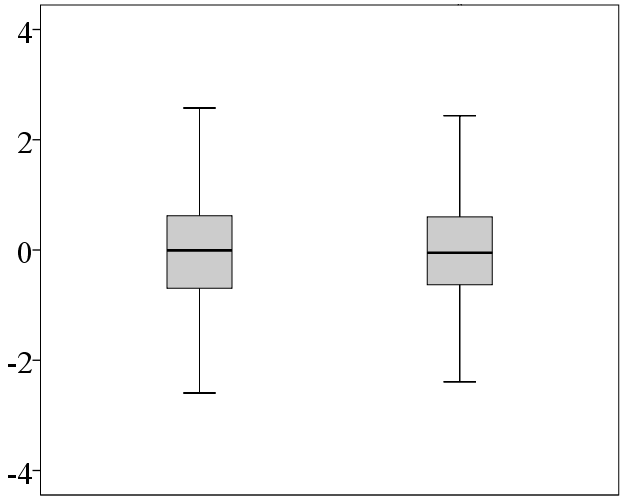

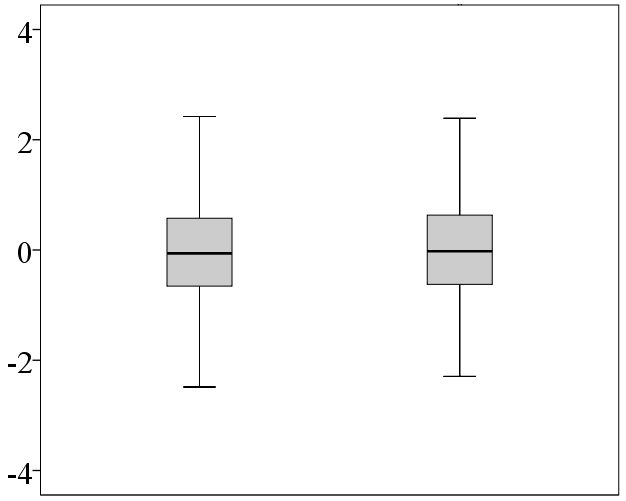

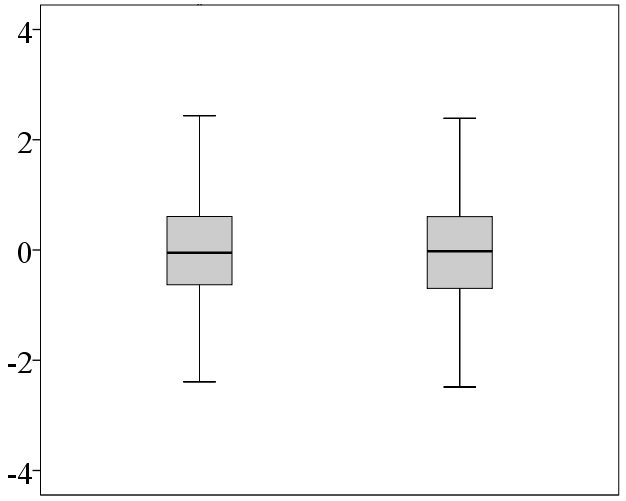

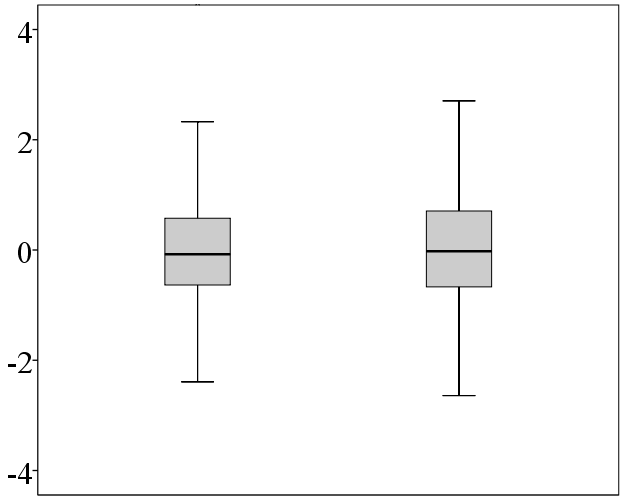


**HR-PRS**

**RMSSD-PRS**

**Controls Current**

**depression/anxiety**

**Controls Current**

**depression/anxiety**

**Controls Current**

**depression/anxiety**
